# Zileuton Alleviates Radiation-Induced Cutaneous Ulcers via Inhibition of Senescence-Associated Secretory Phenotype in Rodents

**DOI:** 10.3390/ijms23158390

**Published:** 2022-07-29

**Authors:** Mineon Park, Jiyoung Na, Seo Young Kwak, Sunhoo Park, Hyewon Kim, Sun-Joo Lee, Won-Suk Jang, Seung Bum Lee, Won Il Jang, Hyosun Jang, Sehwan Shim

**Affiliations:** 1 National Radiation Emergency Medical Center, Laboratory of Radiation Exposure and Therapeutics, Korea Institute of Radiological and Medical Science, Seoul 01812, Korea; minun0215@kirams.re.kr (M.P.); jyna0914@naver.com (J.N.); ksy@kirams.re.kr (S.Y.K.); sunhoo@kirams.re.kr (S.P.); hw0227@kirams.re.kr (H.K.); sjlee@kirams.re.kr (S.-J.L.); wsjang@kirams.re.kr (W.-S.J.); sblee@kirams.re.kr (S.B.L.); zzang11@kirams.re.kr (W.I.J.); 2 Department of Pathology, Korea Cancer Center Hospital, Korea Institute of Radiological and Medical Sciences, Seoul 01812, Korea; 3 Department of Radiation Oncology, Korea Institute of Radiological and Medical Sciences, Seoul 01812, Korea

**Keywords:** cutaneous radiation ulcer, p38, senescence, zileuton, 5-lipoxygenase

## Abstract

Radiation-induced cutaneous ulcers are a challenging medical problem for patients receiving radiation therapy. The inhibition of cell senescence has been suggested as a prospective strategy to prevent radiation ulcers. However, there is no effective treatment for senescent cells in radiation ulcers. In this study, we investigated whether zileuton alleviated radiation-induced cutaneous ulcer by focusing on cell senescence. We demonstrate increased cell senescence and senescence-associated secretory phenotype (SASP) in irradiated dermal fibroblasts and skin tissue. The SASP secreted from senescent cells induces senescence in adjacent cells. In addition, 5-lipoxygenase (5-LO) expression increased in irradiated dermal fibroblasts and skin tissue, and SASP and cell senescence were regulated by 5-LO through p38 phosphorylation. Finally, the inhibition of 5-LO following treatment with zileuton inhibited SASP and mitigated radiation ulcers in animal models. Our results demonstrate that inhibition of SASP from senescent cells by zileuton can effectively mitigate radiation-induced cutaneous ulcers, indicating that inhibition of 5-LO might be a viable strategy for patients with this condition.

## 1. Introduction

Radiation-induced cutaneous ulcers following accidental or intentional acute exposure to high doses of ionizing radiation are a challenging medical problem [1]. Radiation-induced cutaneous ulcers are characterized by the occurrence of unpredictable cycles of inflammation that extend beyond the initial damage, increasing the affected area on the epidermis and deeper tissues, owing to delayed tissue necrosis and repair failure [2,3]. Radiation considerably contributes to inflammation and cell senescence of the investigated tissue, and targeting senescent cells is a potential therapeutic strategy for skin radiation ulcers [4,5,6].

Cell senescence is an evolutionarily conserved state of stable replicative arrest induced by pro-aging stressors, including inflammation [7]. Senescent cells cannot divide, even when stimulated by mitosis, but they maintain metabolic and synthetic activity and show characteristic morphological changes, such as enlargement, flattening, and increased particle size [8]. Furthermore, there is evidence that cell senescence develops a secretory profile composed mainly of inflammatory cytokines. This phenomenon is called senescence-associated secretory phenotype (SASP) [9,10]. Through SASP, senescent cells can contribute to tissue dysfunction by spreading inflammatory factors to the surrounding healthy cells [11]. Radiation has also been reported to affect surrounding non-irradiated cells through communication with irradiated cells via SASP [12,13,14]. Therefore, we hypothesized that inflammatory factors in SASP are involved in radiation-induced cell senescence, and inhibition of SASP may represent a promising strategy to limit the development of cutaneous radiation ulcers by preventing cell senescence.

The understanding of SASP regulation in senescent cells is incomplete; however, 5-lipoxygenase (5-LO) and cyclooxygenase-2 (COX-2) are upregulated in senescent cells [15,16,17] and are known to regulate inflammatory cytokines through eicosanoid synthesis [18,19]. Leukotrienes (LT) and prostaglandins (PG) are two classes of eicosanoids generated by the metabolism of arachidonic acid via the 5-LO and COX-2 pathways, respectively [20,21]. Recent reports demonstrated that senescence-associated LT release drives pulmonary fibrosis, eliminating senescent cells ameliorates these effects [10,22], and that the synthesis of PG is essential to reinforce senescence-associated cell cycle arrest [23]. Activation of p53 signaling is also associated with cellular senescence [16,21,24]. COX-2 inhibitors have been demonstrated to have no beneficial effects on survival or regeneration after radiation injury [25]. However, whether 5-LO inhibition can prevent radiation-induced cutaneous ulcers remains unclear.

In the present study, we show that irradiation induces 5-LO expression, which is followed by the development of senescence in human dermal fibroblasts and skin tissues of irradiated mice. Moreover, treatment with zileuton, a 5-LO inhibitor, significantly inhibited SASP production and prevented cellular senescence through p38 phosphorylation, resulting in the attenuation of cutaneous ulcers. Our data suggest that zileuton treatment after irradiation might be a potential treatment strategy for patients with radiation-induced cutaneous ulcers and other possible tissue damage.

## 2. Results

### 2.1. Radiation-Induced SASP Triggered Senescence in Adjacent Normal Cells

**Figure 1:**
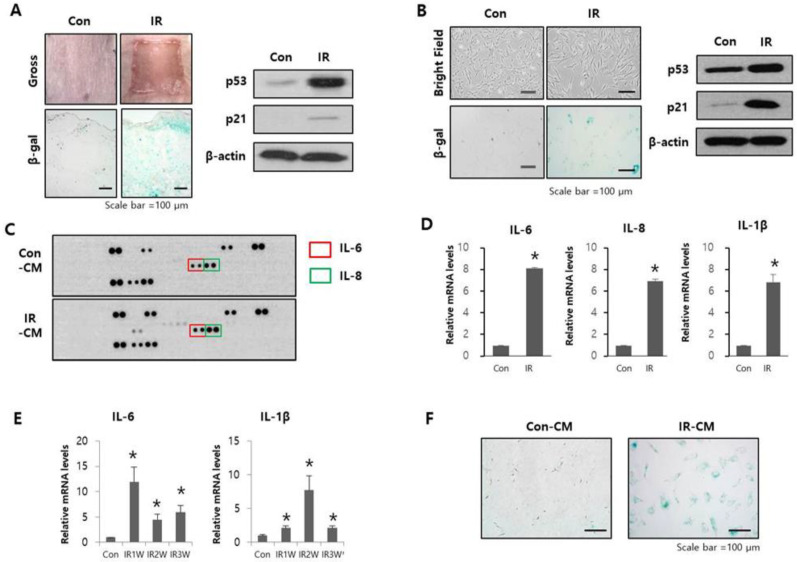
The effects of radiation-induced SASP on adjacent cells and radiation ulcers. (**A**) Representative gross and SA-β-gal staining images (**left**), as well as p53 and p21 expression levels (**right**), in mouse skin 10 days after radiation exposure. Three mice per group were used. (**B**) Representative cell morphological and SA-β-gal staining images (**left**), as well as p53 and p21 expression levels (**right**), in HDFs after radiation exposure. (**C**) Cytokine screening in conditioned medium from control HDFs (Con-CM) and conditioned medium from irradiated HDF (IR-CM). (**D**) mRNA levels of IL-6, IL-8, and IL-1β in HDFs after radiation exposure. Data are presented as mean ± standard deviation with statistical significance set at * *p* < 0.05 vs. control group (Con). (**E**) mRNA levels of IL-6 and IL-1β in mouse skin after radiation exposure. Data are presented as mean ± standard error, with statistical significance set at * *p* < 0.05 vs. control group (Con). Six mice per group were used. (**F**) Representative SA-β-gal staining images in HDFs cultured in Con-CM and IR-CM for 3 days.

To investigate whether SASP is related to cell senescence in radiation-induced cutaneous ulcers, we established a radiation-induced cutaneous ulcer mouse model by high-dose radiation exposure (40 Gy). The irradiated mouse skin revealed desquamation 10 days after irradiation, which resulted in ulceration of the skin (Figure 1A). In irradiated skin sections, we observed an increase in SA-β-gal activity in the dermis 10 days after radiation exposure (Figure 1A). Similarly, we observed increased levels of the senescence markers p53 and p21 in the dermal layer after radiation (Figure 1A). These results revealed that senescent cells accumulated in the dermal layer after radiation exposure and may play a critical role in promoting radiation-induced cutaneous ulcer formation.

As dermal fibroblasts are the major population of cells in the dermal layer, we tested whether radiation induced senescence and released SASP factors in HDFs cells. After 10 Gy radiation exposure, HDFs exhibited larger, overextended terminal branches and increased β-gal activity compared to non-irradiated cells (Figure 1B). We also observed a remarkable increase in p53 and p21 expression (Figure 1B).

Next, we performed secreted cytokine screening using CM from HDF-irradiated (IR-CM) or non-irradiated (Con-CM) cells and detected several upregulated cytokines, including IL-6 and IL-8, in IR-CM compared to Con-CM (Figure 1C). We also analyzed the expression levels of the SASP genes in irradiated HDFs and skin tissues. The mRNA expression of interleukins (IL-6, IL-8, and IL-1β) was significantly upregulated in irradiated HDFs compared non-irradiated HDF cells and tissues (Figure 1D). IL-6 and IL-1β, mRNA levels were also confirmed to be significantly increased in our mouse model (Figure 1E).

To explore whether SASP released from senescent cells by irradiation induces cell senescence in adjacent cells, we treated unirradiated HDF cells with IR-CM and found that it enhanced SA-β-gal activity compared to Con-CM treatment (Figure 1F). These findings suggest that SASP from irradiated cells could induce cellular senescence in adjacent cells and that inhibiting SASP may play a critical role in decreasing senescent cell accumulation.

### 2.2. Radiation Increases 5-LO Expression

**Figure 2:**
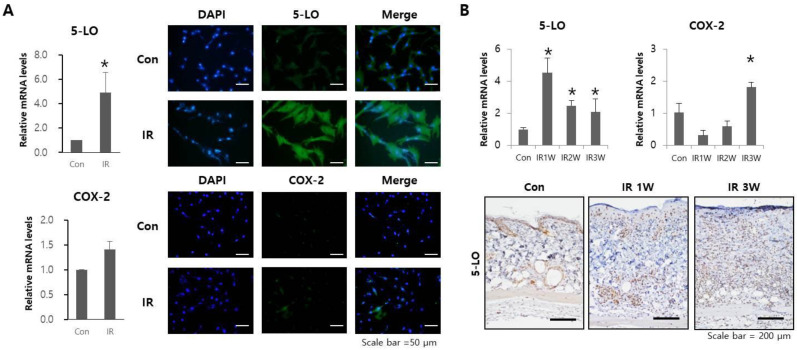
Expression of 5-LO and COX-2 in HDFs and mouse skin tissue after radiation exposure. (**A**) mRNA expression levels and representative immunostaining images of 5-LO (**top**) and COX-2 (**bottom**) in HDFs after radiation exposure. Data are presented as mean ± standard deviation, with statistical significance set at * *p* < 0.05 vs. control group (Con). (**B**) mRNA expression levels of 5-LO and COX-2 (**top**), as well as representative immunostaining images of 5-LO (**bottom**) in mouse skin after radiation exposure. Data are presented as mean ± standard error, with statistical significance set at * *p* < 0.05 vs. control group (Con). Six mice per group were used.

According to recent publications, lipid mediator groups causing inflammation could contribute to SASP production [22,26]. Thus, we analyzed the expression of 5-LO and COX-2, which are the lipid mediators causing inflammation [20] after radiation exposure in HDFs and skin tissues of rodent models. After 10 Gy irradiation, the mRNA and protein expression of 5-LO in HDFs was significantly increased compared to that of COX-2 (Figure 2A). The dorsal skin of mice was exposed to 40 Gy to evaluate the expression of 5-LO and COX-2 in the skin tissue. The 5-LO level in mice exposed to radiation peaked at 1 week and decreased thereafter but remained higher than that of the control mice (Figure 2B). Moreover, the pattern of IL-6 expression was similar to that of 5-LO in irradiated skin (Figure 1D). However, the expression of COX-2 increased after 4 weeks in the skin of treated mice relative to that in control mice (Figure 2B). These results suggest that 5-LO may be involved in cutaneous response to irradiation.

### 2.3. Radiation-Induced 5-LO Promotes Cell Senescence and SASP

**Figure 3:**
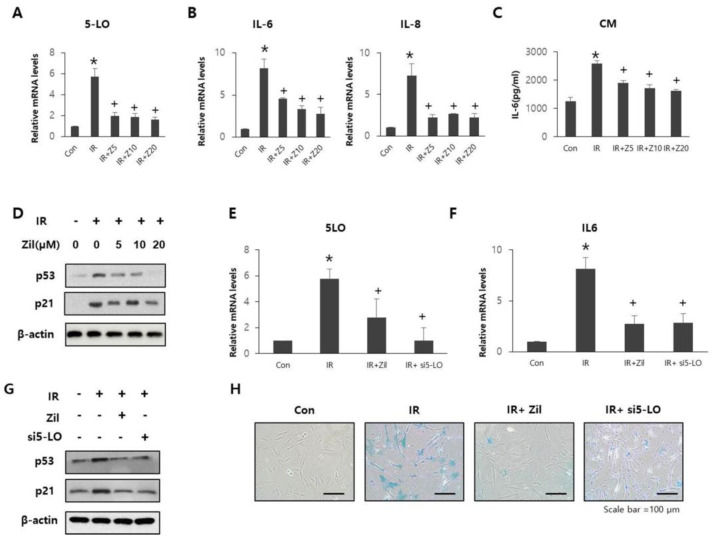
Attenuation of radiation-induced cell senescence and SASP through 5-LO inhibition. (**A**–**D**) HDFs were cultured with various concentrations of zileuton (0, 5, 10, or 20 μM) for 24 h after radiation exposure (*Con:* control, *IR:* irradiation group, *Z5:* 5 μΜ zileuton treatment, *Z10:* 10 μΜ zileuton treatment, *Z20:* 20 μΜ zileuton treatment). (**A**,**B**) mRNA expression levels of 5LO, IL-6, and IL-8 in HDFs. Data are presented as mean ± standard deviation with statistical significance set at * *p* < 0.05 vs. control group (Con), ^+^ *p* < 0.05 vs. irradiated group (IR). *n* = 3; ANOVA (analysis of variance). (**C**) Secreted IL-6 levels in CM from HDFs. Data are presented as means ± standard deviation, with statistical significance set at * *p* < 0.05 vs. control group (Con), ^+^ *p* < 0.05 vs. irradiated group (IR). *n* = 3; ANOVA (analysis of variance). (**D**) p53 and p21 expression levels in HDFs. (**E**–**H**) HDFs were treated with zileuton (Zil) or transfected with the siRNA targeting si5-LO (si5-LO). (**E**,**F**) mRNA expression levels of 5-LO and IL-6 in HDFs. Data are presented as mean ± standard deviation, with statistical significance set at * *p* < 0.05 vs. control group (Con), ^+^ *p* < 0.05 vs. irradiated group (IR). *n* = 3; ANOVA (analysis of variance). (**G**) p53 and p21 expression levels in HDFs. (**H**) Representative SA-β-gal staining images in HDFs.

Next, we investigated the role of radiation-induced 5-LO in SASP and cellular senescence by treating cells with zileuton and silencing 5-LO. HDFs were cultured in the presence of various concentrations of zileuton (0, 5, 10, or 20 μM) for 24 h after irradiation. Zileuton treatment at concentrations up to 20 μM significantly reduced 5-LO (Figure 3A) and SASP levels (Figure 3B,C). In particular, 20 μM zileuton significantly inhibited the expression of senescence markers (Figure 3D). Therefore, 20 μM of zileuton was chosen for all subsequent experiments.

Figure 3E shows that the mRNA expression of 5-LO and IL-6 was reduced in both the IR+Zil and IR+si5LO groups (Figure 3F). Moreover, the expression of senescence factors (Figure 3G) and B-gal activity (Figure 3H) were inhibited in the IR+Zil and IR+si5-LO groups compared to those in the IR group (Figure 3H). Taken together, these results indicate that after irradiation, 5-LO can play an important role in promoting SASP and senescence in HDFs, and 5-LO inhibition effectively attenuates the induction of SASP and senescence in irradiated HDFs.

### 2.4. Zileuton Prevents Cell Senescence and SASP through p38 Phosphorylation

**Figure 4:**
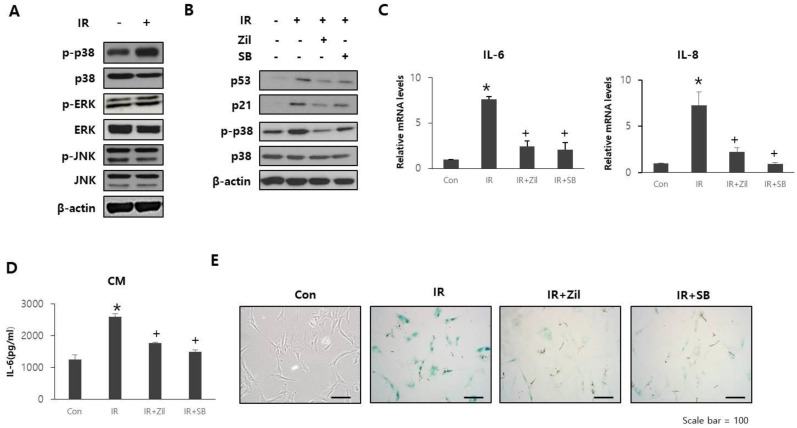
Effects of p38 inhibition on radiation-induced senescence. (**A**) HDF cells were exposed to 10 Gy radiation. After 24 h, levels of p-ERK, ERK, p-p38, p38, p-JNK, and JNK were assessed by Western blotting. (**B**–**E**) HDF cells were irradiated and further treated with zileuton (Zil) or SB203580 (SB). (**B**) p53, p21, p-p38, and p38 expression levels in HDFs. (**C**) IL-6 and IL-8 mRNA levels in HDFs. Data are presented as mean ± standard deviation, with statistical significance set at * *p* < 0.05 vs. control group (Con), ^+^ *p* < 0.05 vs. irradiated group (IR). *n* = 3; ANOVA (analysis of variance). (**D**) Secreted IL-6 levels in CM from HDFs. Data are presented as mean ± standard deviation. * *p* < 0.05 vs. control group (Con), ^+^ *p* < 0.05 vs. irradiated group (IR). *n* = 3; ANOVA (analysis of variance). (**E**) Representative SA-β-gal staining images in HDFs.

To study the potential mechanisms mediating the protective effects of zileuton in cell senescence, we explored whether zileuton treatment of HDFs results in the activation of molecular events associated with senescence. However, canonical DNA damage response signaling is not sufficient for SASP induction, whereas p38, a member of the mitogen-activated protein kinase (MAPK) family, is one of the regulators of SASP induction [27]. In addition, p38 is activated following radiation exposure [26,28] and upregulates specific cytokines, such as IL-6 and IL-8, in some biological contexts [29,30]. As shown in Figure 4A, after exposure to radiation, p38 phosphorylation increased in HDFs. To determine the role of p38 in zileuton-induced inhibition of cell senescence, we compared the effects of zileuton and SB203580, a p38 inhibitor, on HDFs. Zileuton treatment inhibited the increased phosphorylation of p38, p53, and p21 in irradiated HDFs (Figure 4B). Moreover, SB203580 treatment resulted in a decrease in p53 and p21 expression levels in irradiated HDFs (Figure 4B). Furthermore, SB203580 treatment significantly inhibited the SASP levels (Figure 4C,D) and β-gal activity (Figure 4E) in irradiated HDFs following zileuton treatment. These results suggest that the zileuton-induced inhibition of cell senescence and SASP are both p38-dependent.

### 2.5. Zileuton Attenuates Radiation-Induced Cutaneous Ulcers

To verify the effects of zileuton on radiation-induced cutaneous wounds, we compared the gross and histological characteristics of the skin of zileuton-treated and control mice after 40 Gy irradiation.

**Figure 5:**
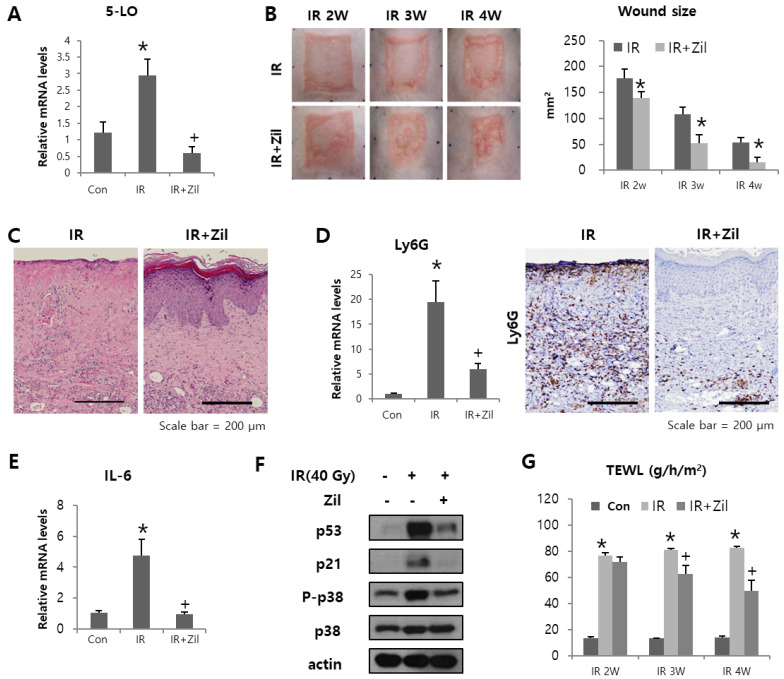
Effect of zileuton in a mouse model of radiation skin injury. Six mice per group were used. (**A**) 5-LO mRNA levels in mouse skin in control (Con), irradiated (IR), and zileuton-treated irradiated (IR+Zil) mice. Data are presented as mean ± standard error, with statistical significance set at * *p* < 0.05 vs. control group (Con), ^+^ *p* < 0.05 vs. irradiated group (IR). (**B**) Representative gross images of mouse skin and quantification of wound size. Data are presented as mean ± standard error, with statistical significance set at * *p* < 0.05 vs. irradiated group (IR). (**C**) Hematoxylin and eosin (H&E) staining of mouse skin. (**D**) mRNA levels (**left**) and representative immunostaining images (**right**) of Ly6G in mouse skin. Data are presented as mean ± standard error, with statistical significance set at * *p* < 0.05 vs. control group (Con), ^+^ *p* < 0.05 vs. irradiated group (IR). (**E**) mRNA levels of IL-6 were measured by qRT-PCR. Data are presented as mean ± standard error, with statistical significance set at * *p* < 0.05 vs. control group (Con), ^+^ *p* < 0.05 vs. irradiated group (IR). (**F**) Western blot analysis of p53, p21, p-p38, and p38 expression levels of mouse skin. (**G**) Transdermal water loss values of mouse skin. Data are presented as mean ± standard error, with statistical significance set at * *p* < 0.05 vs. control group (Con), ^+^ *p* < 0.05 vs. irradiated group (IR).

The areas of irradiation were topically applied with either a 100 µL 0.9% NaCl solution (IR) or 200 μΜ Zileuton. In the zileuton treatment group, the radiation-induced increase in 5-LO levels was significantly inhibited (Figure 5A). Digital photographs chronicled the progress made in wound resolution in the IR and zileuton groups. All irradiated sites exhibited skin desquamation from 2 weeks after radiation exposure (Figure 5B). However, the wound size in zileuton-treated mice was smaller than that in IR mice, and unlike the open wounds of IR mice, the wounds of zileuton-treated mice were nearly closed at 4 weeks (Figure 5B). Quantification of active wound dimensions confirmed significantly faster wound resolution in the zileuton (vs. IR) group at all time points (Figure 5B). These results indicate that topically applied zileuton promotes regeneration of cutaneous radiation ulcers.

The extent of skin regeneration was assessed by microscopy. Photomicrographs of histological preparations indicated that after zileuton treatment, epidermal thickness and layering were more aligned within normal skin at 4 weeks, surpassing the IR group in this regard (Figure 5C). Along with improved closure, zileuton-treated skin had reduced infiltrated neutrophils compared with the IR group. There was a significant increase in Ly6G (neutrophil marker) in the irradiated group, with an increased reduction in the zileuton treatment group at 4 weeks (Figure 5D). Neutrophil infiltration causes necrosis and ulceration in skin diseases. These results indicate a role of 5-LO in the control of neutrophil recruitment in irradiated skin.

As zileuton has been shown to reduce SASP production and cell senescence via p38 in vitro, we next evaluated the expression of SASP and cell senescence markers that could interfere with the outcome of wound healing. There was a remarkable increase in the expression of IL-6, p38, p53, and p21 in the IR group compared with that in the control group (Figure 5E,F). This expression level was maintained at very low levels in the zileuton treatment group.

Epidermal barrier function provides an independent and objective parameter of disease severity in various skin injuries. TEWL is a physiological property that reflects the skin barrier efficiency. TEWL measurements allowed us to compare the reconstitution of barrier function and maturation of wounds in the IR and zileuton treatment groups over time. The TEWL values in the zileuton treatment group were lower than those in the IR group. Specifically, the zileuton treatment group exhibited a nearly linear decline in TEWL values for 2 weeks, whereas values in the IR group did not decrease (Figure 5G).

## 3. Discussion

In a previous report, moderate to severe skin damage was observed in 85% of cancer patients who received radiation therapy [31]. Radiation-induced ulcers can reduce the quality of patients’ life because it has been clearly documented that radiation-induced ulcers cannot heal by themselves [32]. Therefore, the management of radiation-induced ulcers to reduce the complications of radiation therapy has emerged as a clinically important issue.

In the present study, we provide the first evidence of the involvement of 5-LO in radiation-induced SASP in both human fibroblasts and mouse skin influencing radiation ulcers. Radiation ulcers caused by ionizing radiation can damage the skin or other organs. However, the mechanisms underlying the unpredictable and uncontrolled extension of radiation ulcers are not completely understood. Recent evidence suggests that senescent cells that accumulate in irradiated skin accelerate the development of radiation ulcers. During their accumulation, senescent cells secrete a series of factors called SASP, which promotes the spread of senescence to other cells and cause tissue dysfunction [33]. Here, we found that the expression of SASP and accumulation of senescent cells were increased in radiation ulcers and that SASP released from irradiated cells accelerated senescence in adjacent cells in vitro. This led us to hypothesize that preventing SASP production may represent a prospective strategy for mitigation of radiation ulcers.

As dermal fibroblasts appear to be the main population of senescent cells in radiation ulcers, we analyzed cytokine release in a cell culture medium using a cytokine array and mRNA expression in irradiated HDFs using qRT-PCR. The results revealed that SASP, comprising multiple characteristic factors, including pro-inflammatory cytokines, was markedly upregulated in irradiated HDFs. IL-6, a pleiotropic proinflammatory cytokine, is one of the most prominent cytokines in the SASP. It has been reported that IL-6 correlates with DNA damage and stress-induced senescence in various type of cells [13,34,35,36]. Additionally, it appears that senescent cells directly affect neighboring normal cells through IL-6 expression [37].

Here, we showed that senescent cells synthesize 5-LO, a key enzyme in proinflammatory cytokine production. The mRNA level of 5-LO increased significantly after radiation exposure in vitro and in vivo. Furthermore, 5-LO gene knockdown effectively prevented cellular senescence and SASP production. Interestingly, *COX-2* was not overexpressed in senescent cells. Therefore, focusing on the 5-LO pathway is a suitable strategy to prevent senescence, and the inhibition of 5-LO is useful for mitigating radiation ulcers.

In this study, we observed that zileuton prevented radiation-induced cell senescence and SASP. These beneficial effects are achieved by inhibiting the p38-dependent pathway. Our results showed that radiation exposure enhanced p-p38, p53, and p21 protein levels and that p38 is involved in radiation-induced, 5-LO-mediated cell senescence and SASP production. In a recent study, 5-LO was found to regulate cell senescence via p53 activation [16,38]. In our study, we further identified that 5-LO can activate p53 through the p38 pathway, thereby promoting cell senescence.

In support of our findings, we observed that topical zileuton application enhanced healing of cutaneous radiation ulcers in a mouse model. Zileuton treatment effectively inhibited the increase in 5-LO expression and inflammatory cell infiltration. The wound caused by epithelium desquamation was closed faster in the zileuton group than in the IR group. Among the numerous SASP factors upregulated in senescent skin by radiation exposure, the expression patterns of IL-6 were similar to those of 5-LO, and zileuton treatment effectively inhibited the increase in IL-6. We also measured p38 and senescence markers (p53 and p21) that are involved in 5-LO-mediated radiation senescence. The increased phosphorylation of p38 and senescence markers in the IR groups was effectively inhibited by zileuton treatment. These results are consistent with those of our in vitro experiments. Currently, various precautionary methods and therapies, such as anti-inflammatory drugs, growth factors, and steroid creams, are used to treat cutaneous radiation ulcers, although the clinical effects of these therapies is poor, and side effects occur [3]. Zileuton is an orally administered and FDA-approved drug for the treatment of asthma that alleviates disease symptoms by suppressing 5-LO. Additionally, zileuton was used in a trial for atopic dermatitis in a pilot study [39] and has an excellent preclinical GI pharmacological safety profile [40]. Hence, zileuton treatment attenuates radiation ulcers, and there is considerable potential to treat radiation ulcers by developing safe and effective drugs that inhibit SASP.

In summary, our study demonstrated that senescent cells persist in radiation ulcers and that 5-LO regulates SASP and cell senescence through the p38 pathway. Moreover, inhibition of SASP secretion in senescent cells by zileuton can effectively mitigate this painful side effect. Furthermore, our results indicate that inhibiting SASP release from senescent cells is a potential therapeutic method to mitigate radiation ulcers.

## 4. Materials and Methods

### 4.1. Cell Culture & Irradiation

Newborn human foreskin fibroblasts (HDFs) were purchased from GlobalStem, Inc. HDFs were maintained in Dulbecco’s Modified Eagle Medium (DMEM; Gibco, Grand Island, NY, USA) containing 10% (*v*/*v*) fetal Bovine serum (FBS, Gibco) and 1% (*v*/*v*) antibiotics. HDF cells were irradiated with 10 Gy using a 137Cs γ-ray source (Atomic Energy of Canada, Ltd., Chalk River, ON, Canada) at a dose rate of 3.25 Gy/min and treated with zileuton (Cayman Chemical, Ann Arbor, MI, USA) for 5-LO inhibition or SB203580 (Calbiochem^®^, San Diego, CA, USA) for p38 inhibition.

### 4.2. Conditioned Medium (CM)

HDFs were seeded into 100 mm culture dishes at a density of 5 × 10^5^ cells/mL. To obtain CM, HDFs were irradiated and further treated with vehicle, Zileuton, or SB203580. After 24 h, the media were harvested and filtered. Control cells were not irradiated and were subjected to the same chemical treatment.

### 4.3. Cytokine Array and Enzyme-Linked Immunosorbent Assay (ELISA)

The cytokine array was performed using conditioned media from control (Con) and irradiated (IR) groups. CM was analyzed using a Proteome Profiler™ human cytokine array kit (R&D Systems, Minneapolis, MN, USA) according to the manufacturer’s instructions. The concentration of the human inflammatory cytokine IL-6 in the CM obtained from HDF cells was measured using an enzyme-linked immunosorbent assay (ELISA) kit (R&D Systems, Minneapolis, MN, USA) according to the manufacturer’s protocol.

### 4.4. Small Interfering RNA Transfection

Human 5-LO-specific siRNA oligonucleotides were purchased from Santa Cruz Biotech. Scrambled-sequence oligonucleotides were used as transfection controls. RNAi MAX (Thermo Fisher Scientific, Waltham, MA, USA) was used to transfect oligonucleotides according to the manufacturer’s instructions.

### 4.5. Animal Irradiation

Male SKH-1 mice (6–8 weeks) were purchased from DooYeol Biotech (Seoul, Korea). All animal procedures were approved by the Animal Investigation Committee of the Korea Institute of Radiological and Medical Sciences. They was maintained under specific pathogen-free conditions in the KIRAMS animal facility. All mice were housed in a temperature-controlled room with a 12 h light–dark cycle. Animals were anesthetized by intraperitoneal injection of 75 mg/kg of alfaxalone (Alfaxan^®^; Careside, Seongnam-si, Gyeonggi-do, Korea) and 10 mg/kg of xylazine (Rompun^®^, Bayer Korea, Seoul, Korea), and dorsal skin (other parts covered with lead board) was exposed to 40 Gy radiation under anesthesia using an X-RAD 320 instrument (Softex, Goyang-si, Gyeonggi-do, Korea; filter:2 mm AI; 260 kV, 10 mA; 2.0 Gy/min). Dorsal skin was gently stretched and maintained with tape within the irradiation field. The area of the dorsal skin exposed to IR corresponded to a surface measuring 2 × 2 cm. The rest of the mouse body was protected from radiation using 6 mm-thick lead shielding.

To evaluate the involvement of 5-LO in the modulation of the skin response, the irradiated skin area was topically covered with 100 µL of 200 µM zileuton solution, starting on the day of irradiation. Zileuton was applied daily until the day of euthanasia. Aqueous polymeric gel base was prepared by adding propylene glycol (30%), polyethylene glycol (7%), and Na-CMC (1.5%) in water under continuous stirring with a magnetic stirrer until the gel was formed [41]. Then required amount of zileuton was added slowly to the polymeric gel under constant stirring to obtain a homogenous dispersion.

### 4.6. Wound Analysis

Images of the wounds were captured weekly using a digital camera. Wound size was measured by tracing the wound margin and performing calculations using image analysis software (i-solution^TM^, IMT, Daejeon, Korea).

### 4.7. Transepidermal Water Loss Analysis

Transepidermal water loss (TEWL) was measured using a Tewameter TM 300 (Courage and Khazaka Electronic GmbH, Köln, Germany) according to the manufacturer’s protocol. TEWL was measured in the dorsal skin of irradiated mice. The measurements were performed under controlled conditions, including constant relative humidity and room temperature.

### 4.8. Histological Examination

Excised skin patches were fixed in 4% paraformaldehyde, embedded in paraffin, sectioned at 4 µm, and stained with H&E for microscopic examination.

### 4.9. Immunostaining

HDF cell monolayers on coverslips were harvested, and immunocytochemistry analysis was performed. Cells were fixed with 4% paraformaldehyde, blocked with 3% BSA, permeabilized with 0.1% Triton x-100 in 1% BSA for 30 min at room temperature, and incubated overnight at 4 °C with the primary antibodies specific to 5-LO (Santa Cruz, CA, USA) and COX-2 (Santa Cruz, CA, USA). Next, the cells were incubated for 1 h at room temperature with Alexa Fluor 488 (green)-conjugated anti-mouse IgG (Thermo Fisher Scientific) as secondary antibodies. After washing with DPBS, the cells were counterstained with DAPI and mounted using a mounting solution.

For tissue immunohistochemistry, antigen retrieval was performed by autoclave heating, and endogenous peroxidase activity was blocked with 0.3% H_2_O_2_ in methanol. To assess the expression levels of 5-LO and Ly6G, slides were blocked for 1 h with 10% normal goat serum (Vector Laboratories, Burlingame, CA, USA) and allowed to react with anti-5-LO (Abcam, Cambridge, UK) and anti-Ly6G (Abcam, Cambridge, UK) for 2 h at room temperature, followed by 1 h incubation with the HRP-conjugated secondary antibody (Dako, Carpinteria, CA, USA). The peroxidase reaction was developed using a DAB kit (Dako, Carpinteria, CA, USA) according to the manufacturer’s instructions.

### 4.10. RNA Isolation and Quantitative Real-Time PCR

Total RNA was isolated using TRIzol reagent (Invitrogen, Carlsbad, CA, USA) according to the manufacturer’s instructions. cDNA was synthesized using AccuPower RT premix (Bioneer, Daejeon, Korea). The synthesized cDNA was amplified using a Light Cycler 480 system (Roche, Basel, Switzerland) with specific primers. The expression level of each gene was determined using the ΔΔCt method. The primers used for qRT-PCR are listed in Table 1.

### 4.11. SA-β-Gal Staining

Irradiated and control HDFs were further treated with vehicle, Zileuton, or SB203580 for 24 h. Then, the cells were cultured for 1 day after plating. SA-β-gal staining was performed using an SA-β-gal staining kit (Cell Signaling Technology, Danvers, MA, USA) according to the manufacturer’s instructions. Cells were fixed with 4% paraformaldehyde for 15 min and incubated in SA-β-Gal staining solution (pH 6.0) overnight at 37 °C. For SA-β-Gal activity in the skin, 5 µm frozen sections were fixed with 4% paraformaldehyde for 15 min, washed with PBS, and incubated in SA-β-Gal staining solution (pH 6.0) overnight at 37 °C. Cells stained blue were identified as senescent under a light microscope.

### 4.12. Western Blot

Cells and tissue specimens were homogenized in RIPA lysis and extraction buffer (Thermo Fisher Scientific). Extracted proteins were separated on a 10–12% sodium dodecyl sulfate polyacrylamide gel and transferred to a polyvinylidene difluoride membrane (GE Healthcare, Little Chalfont, UK). Membranes were blocked in 5% skimmed milk for 30 min and rinsed briefly in TBS-T. The following antibodies were used: p21 (Santa Cruz Biotechnology), p53 (Santa Cruz Biotechnology), phospho-p38 (Cell Signaling Technology), p38 (Cell Signaling Technology), phospho-ERK (Cell Signaling Technology), ERK (Cell Signaling Technology), and JNK (Cell Signaling Technology). β-actin (Santa Cruz Biotechnology) was used as a control. Following overnight incubation at 4 °C, membranes were washed and incubated with secondary antibodies for 1 h. The membrane was washed, and proteins were detected using an enhanced chemiluminescence reagent (Pierce, Thermo Fisher Scientific).

### 4.13. Statistical Analysis

Data were analyzed using the GraphPad prism 8 statistical program. The in vitro data were plotted as mean ± standard deviation, and the animal data were plotted as the mean ± standard error. Statistical analyses were performed using one-way analysis of variance (ANOVA) with Tukey’s multiple comparison test. Statistical significance was set at *p* < 0.05.

## Figures and Tables

**Table 1:** Primer sequences for qRT-PCR.

Species	Gene	Forward Primer	Reverse Primer
Mouse	IL-1β	CAGCTCATATGGGTCCGACA	CTGTGTCTTTCCCGTGGACC
	IL-6	AGCCAGAGTCCTTCAGAGAG	GATGGTCTTGGTCCTTAGCC
	COX-2	CCAGCACTTCACCCATCAGTT	ACCCAGGTCCTCGCTTATGA
	5-LO	GCCGGACTGATGTACCTGTT	CGCTTCCGAAGAAGAAGATG
	Ly6G	GAGAGGAAGTTTTATCTGTGCAGC	TCTCAGGTGGGACCCCAATA
	Β-actin	CTTTTCACGGTTGGCCTTAG	CCCTGAAGTACCCCATTGAAC
Human	IL-1β	ATGATGGC TTATTACAGTGGCAA	GTCGGAGATTCGTAGCTGGA
	IL-6	ATGGGAAACAATGTCACGAAC	TGTATTCCGTCTCCTTGGTTC
	IL-8	CTCTTGGCAGCCTTC CTGATT	ACTCTCAATCACTCTCAGTTCT
	5-LO	ACAAGCCCTTCTACAACGACT	AACTGGG CGAGATCCAGCT
	COX-2	GTTCCACCCGCAGTACAGAA	AGGGCTTCAGCATAAAGCGT
	GAPDH	GGACTCATGACCACAGTCCATGCC	TCAGGGATGACCTTGCCCACAG

## Data Availability

Not applicable.
